# Amusements

**Published:** 1890-05

**Authors:** 


### HALL'S
### JOURNAL OF HEALTH

TRUTH DEMANDS NO SACRIFICE; ERROR CAN MAKE NONE.

Vol. 37. MAY, 1890. No. 5.

### AMUSEMENTS.

As surely as the body requires a variety of wholesome food for its proper nourishment, the mind needs diversion in order to maintain itself to the level of its highest capacity. There should be intervals of pleasure; of relaxation from the dull routine of business or professional and mechanical pursuits, such as are to be found in out-door sports, or fireside games.

It is a trite saying that "All work and no play makes Jack a dull boy," and nothing can be truer. It is downright cruelty to withhold from the young those innocent amusements in which they find so much real enjoyment. They should be encouraged and assisted if need be, that the home may be made cheerful and inviting, in a word, the dearest place on earth to them. Do not discourage their play nor check the ring of their merriment, even if it be at times a little boisterous. Laughter is better than physic, for it often does away with its necessity.

It is pitiable to find the home circle composed in part of little half-grown old men and women, whose tender years have been despoiled of their birthright by a sort of moral curb that is tightened with a jerk at every outbreak of rebellious nature. Children reared in this way are sure to be puny and always ailing, like frail exotics rooted in unfriendly soil, and forced into a growth that only half expresses their native loveliness. They may be "pinks of propriety" in unsoiled linen, but better far the careless ways and hearty robustness of childhood, when allowed to follow becomingly, those natural propensities which can only be uprooted by means which cause the flowers of youth to wither and its springs to dry up. Don't be afraid of a little dirt. Mother earth is full of magnetic force which she imparts only by contact, and never tires of health-giving to those who are wise enough to understand her manifold ways. We have often heard the remark by solicitous parents, "Why is it that my children, who have the best of care, in contrast with those who seem to be left to go about unattended and wallow in dirt, in point of health, appear to such disadvantage?"

The remark explains itself. It is precisely this careless freedom to go about with bare feet, build turf play-houses and make mud pies, that gives the one that health and strength which the other lacks. It is, in fact, just the difference between the potted plant of the green-house, and the vigorous out-door shrub, that breathes the air in its freshness, and fills its flowering cups with heavenly rain. One lives with nature and obeys her laws, the other is the unwilling slave of art.

What we have found occasion to say of the need of home amusements for children, applies with equal force to adults, so far, at least, as diversions from plodding every day pursuits, serve as promoters of health. If the women of the household could appreciate how entirely their beauty, and more than all, their strength and vigor, depend upon sunshine and exercise, preferably in the open air, there would be less lounging in easy chairs by the shaded window, and a heartier welcome to out-door pastimes in the garden or among the flower beds, or in lack these, in walks and rambles, where nature is lavish of her charms, or art displays its cunning handiwork.

Then there is the play-house, where, when at the best, moral lessons are inculcated in a manner to instruct as well as to amuse; the opera, whose inconsistencies are lost to view in a flow of delicious harmony; the museum and lecture hall, and a variety of similar entertainments not without their benefits, and above all, the pleasantries of the home, which should never be tyrannized over, or corrupted by the base alloy of business.

We have an acquaintance with a gentleman whose years are verging upon the allotted three score and ten. In stature he is tall, erect, and carries himself with something of a military air ; a more vigorous and healthy person you would not meet with in a day's journey. In early manhood he was employed in an extensive mercantile house in New England's much lauded metropolis, a devotee to business, which absorbed all his attention. Of more than ordinary ability, he gained rapid promotion, and soon found himself the trusty and trusted head of a department, and year in and year out, in the hours of business, he was ever to be found at his post of duty.

After a time it was noticeable that this constant application was slowly but surely undermining our friend's health, and so solicitous of his welfare was his employer, that he insisted upon providing a home for him at his own residence, with no lack of personal comfort; but these attentions availed nothing. Day by day the inroads of disease made steady progress, till at length the youthful sufferer was forced to abandon his place in the busy ranks of commerce, and was thought to be incurable.

In this crisis he aroused himself and took on a resolution which, in his weak and wasted condition, was looked upon as madness ; indeed, a veritable challenge to death. He packed his trunk and set out upon his travels, leaving his sorrowing friends behind, with a no more definite plan than to go somewhere he had never been before, and to keep going from place to place amid new scenes, by easy stages, and frequent rests, no matter where. In due course, he brought up at Niagara Falls, where he became absorbed with the wonders which presented themselves upon every hand, making new acquaintances, and exciting their sympathy as one doomed to an untimely end.

But in all and through all, our friend never lost courage or shrank from the regimen he had prescribed for himself. His habits were regular, his diet simple, with a modicum of reviving stimulants. Every local change presented some new attraction that drew him further and further away from himself, till his disease ceased to be his pet, becoming more and more subordinated to health giving influences, till at length he surprised his friends by appearing among them as one raised from the dead. This was more than forty years ago.

The *Boston Herald*, of a recent date, gives an account of an old gentleman who recovered by precisely the same means, from a low stage of consumption, after being so reduced as to be scarcely able to walk.

The secret of these remarkable cures lies not in drugs, but in sunshine, pure country air, healthy diet, and judicious exercise, with frequent changes of scene, that diverted the mind of its brooding cares, and drew it away from its sorrows.

"Better to hunt in fields for health unbought,
Than fee the doctor for a nauseous draught,
The wise for cure on exercise depend;
God never made his work for man to mend."

